# Supplementary material for: Molecular and anatomical roadmap of stroke pathology in immunodeficient mice
Source: Front Immunol. 2022 Dec 9;13:1080482. doi: 10.3389/fimmu.2022.1080482 (PMC9785704; doi:10.3389/fimmu.2022.1080482)
Supplement: Supplementary file 1 [file DataSheet_1.pdf]

Supplementary Material

Molecular and anatomical roadmap of stroke pathology in immunodeficient mice

Rebecca Z Weber[1,2], Geertje Mulders[3], Patrick Perron[1], Christian Tackenberg[1,2], Ruslan Rust[1,2,*]

Affiliation

[1] Institute for Regenerative Medicine, University of Zurich, 8952 Schlieren, Switzerland,

[2] Neuroscience Center Zurich, University of Zurich and ETH Zurich, Zurich, Switzerland

[3] Department of Health Sciences and Technology, ETH Zurich, Zurich, Switzerland

* Correspondence

Ruslan Rust
Institute for Regenerative Medicine (IREM)
University of Zurich, Campus Schlieren
Wagistrasse 12
8952 Schlieren / Zurich, Switzerland
ruslan.rust@irem.uzh.ch, +41 44 63 53215

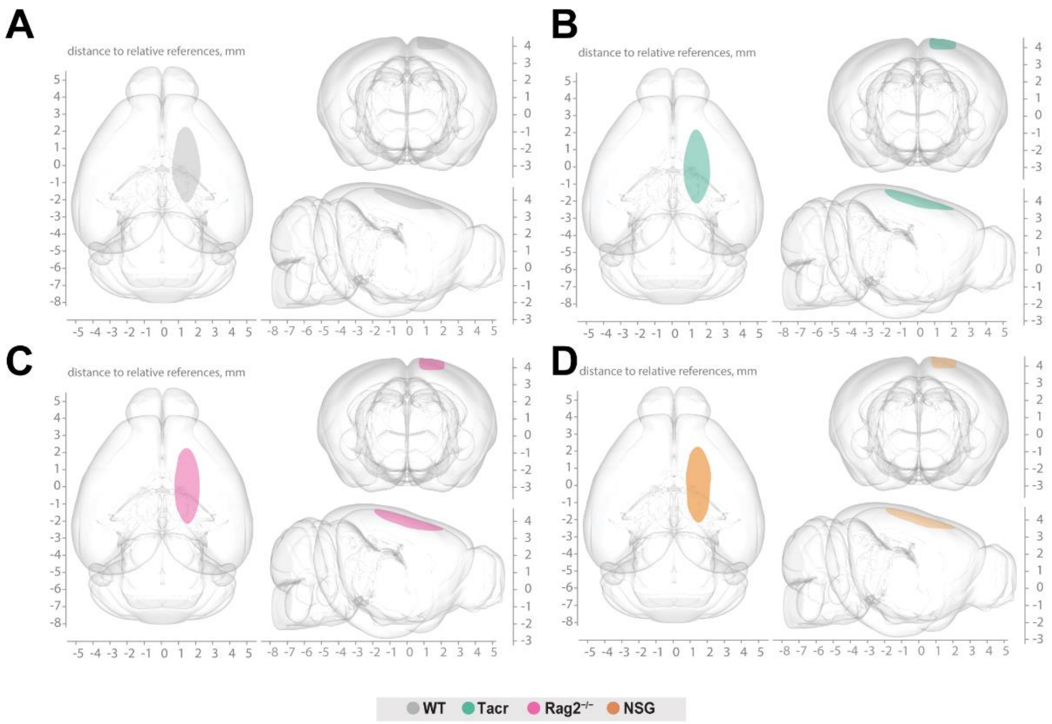

Suppl. Fig. 1: Stroke volume and location. 3D reconstruction of stroke location within a brain template from three perspectives of (A) C57BL/6J wildtype (WT), (B) Tacrolimus immunosuppressed wildtype (WT-Tacr), (C) recombination activating gene 2 deficient mice ($Rag2^{-/-}$) and (D) NOD scid gamma mice (NSG).

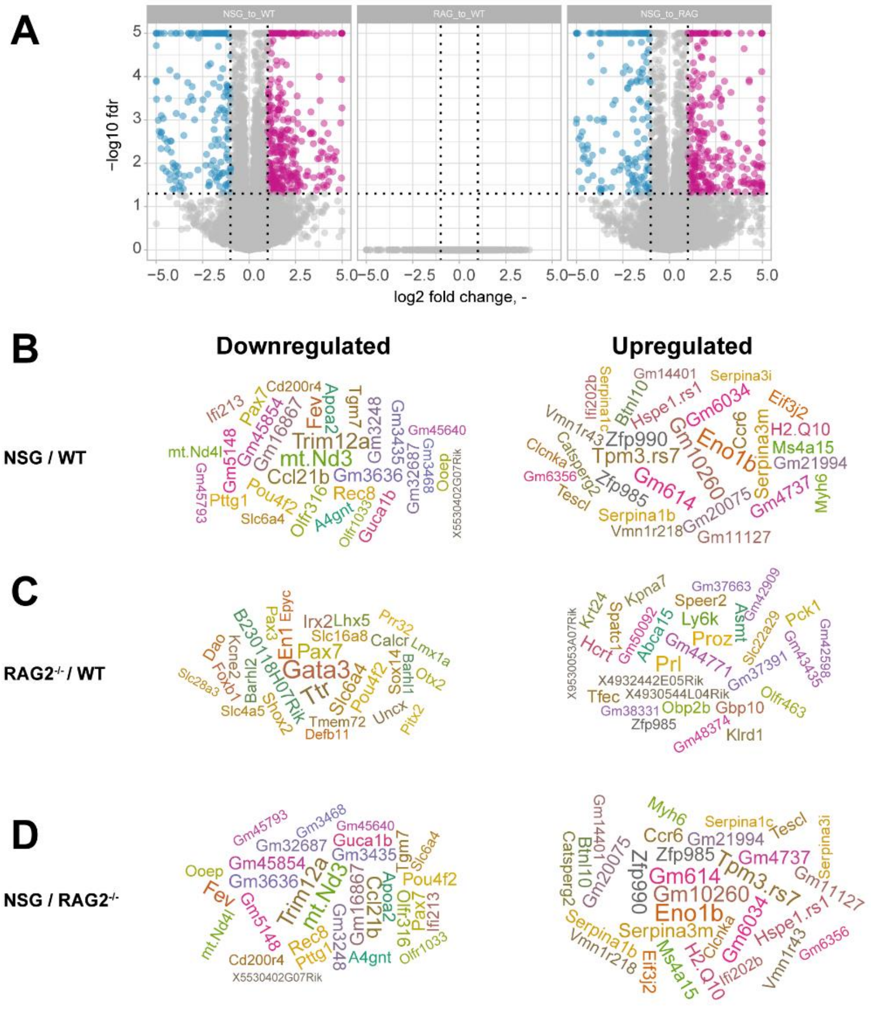

Suppl. Fig. 2: Gene expression differences of non-stroked WT and non-stroked immunosuppressed mice. (A) Volcano plot of non-stroked NSG mice to non-stroked WT (left) non-stroked Rag2$^{-/-}$, to non-stroked WT (middle) and non-stroked NSG to non-stroked Rag2$^{-/-}$ (right) (B) List of top 30 upregulated and downregulated genes after stroke in (B) non-stroked NSG mice to non-stroked WT (C) non-stroked Rag2$^{-/-}$, to non-stroked WT and (D) non-stroked NSG to non-stroked Rag2$^{-/-}$. Font size represents the strength of upregulation/downregulation.

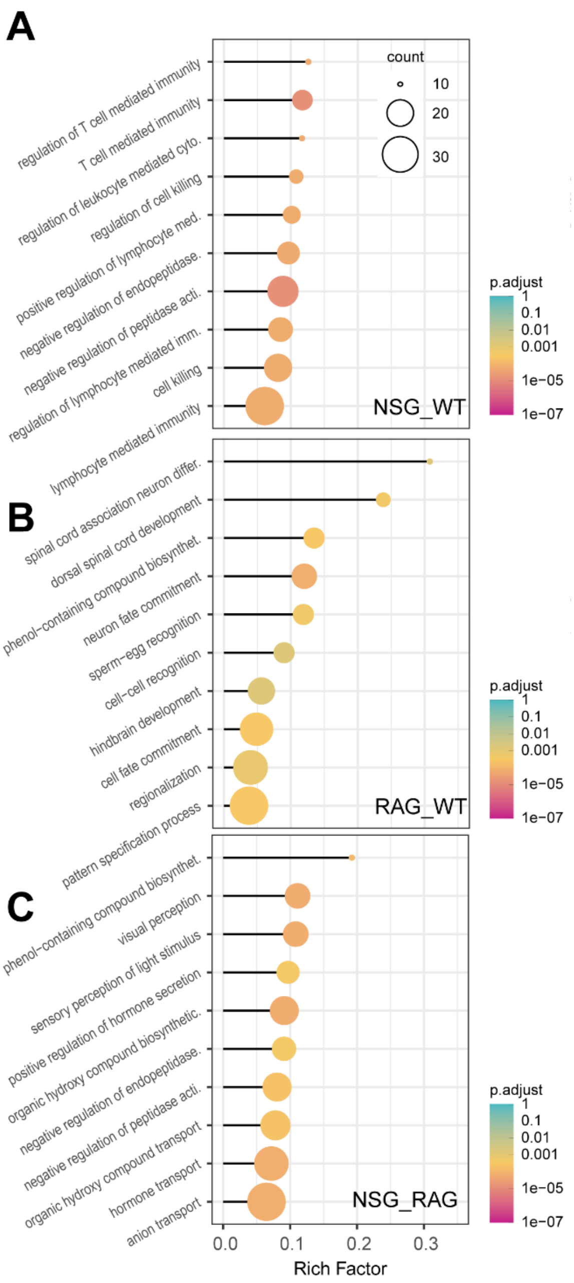

Suppl. Fig. 3: Gene set enrichment analysis in stroked mice. Lollipop plot and of top 10 pathways enriched in (A) non-stroked NSG mice to non-stroked WT (B) non-stroked Rag2$^{-/-}$, to non-stroked WT and (C) non-stroked NSG to non-stroked Rag2$^{-/-}$. Size of dots represents the number of genes in the pathway and color of dots represents adjusted p value.

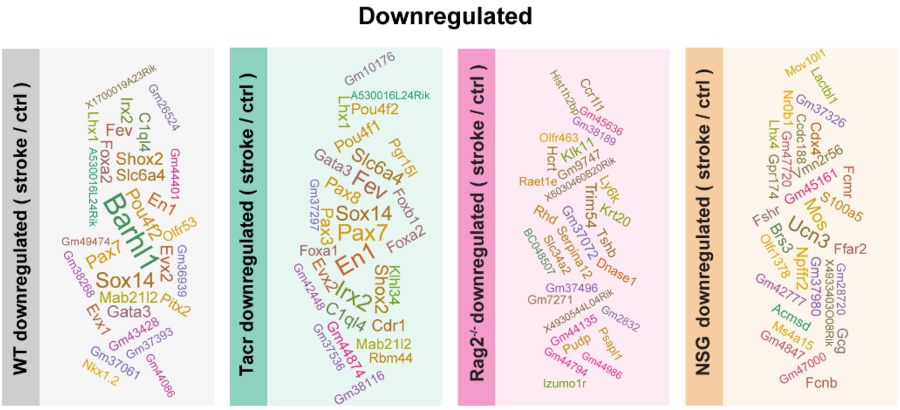

Suppl. Fig. 4: Downregulated genes after stroke in the respective group of immunodeficient mice. Word cloud plot of the top 30 downregulated genes after stroke in the respective group of immunodeficient mice compared to the control group, font size represents the strength of downregulation.

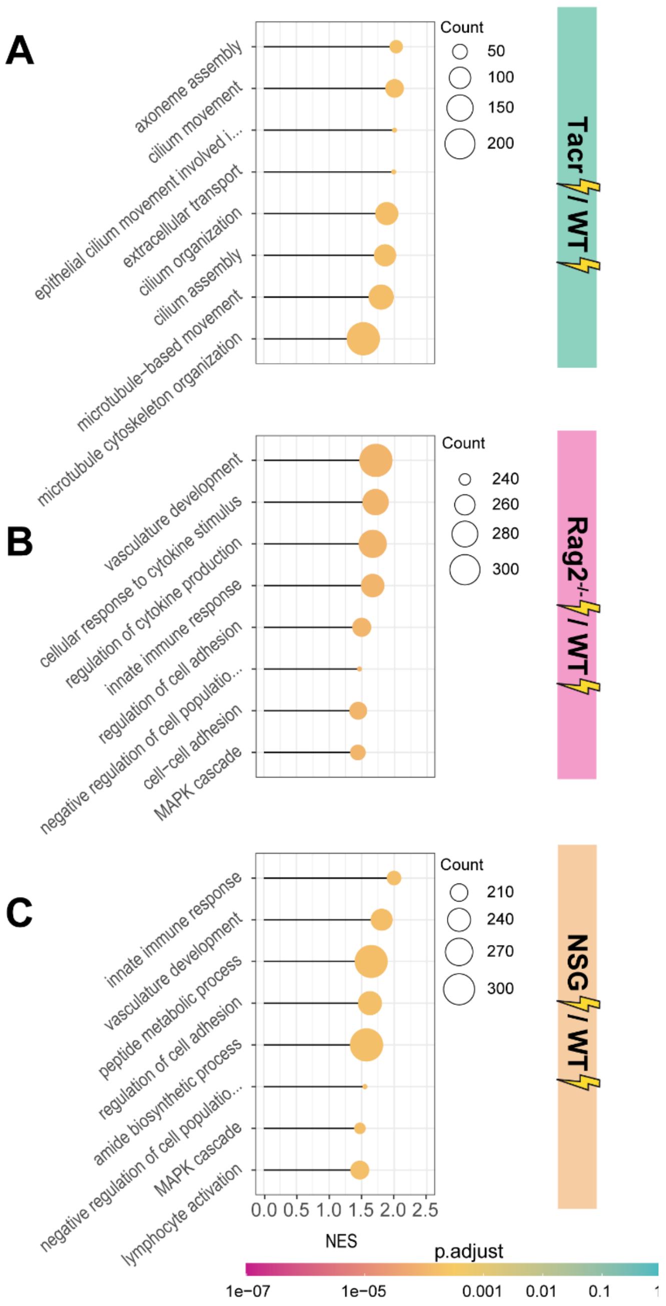

Suppl. Fig. 5: Gene set enrichment analysis in stroked mice. Lollipop plot and of top 10 pathways enriched in (A) stroked WT-Tacr mice to stroked WT (B) stroked Rag2$^{-/-}$, to stroked WT and (C) stroked NSG to stroked WT$^{-/-}$. Size of dots represents the number of genes in the pathway and color of dots represents adjusted p value.

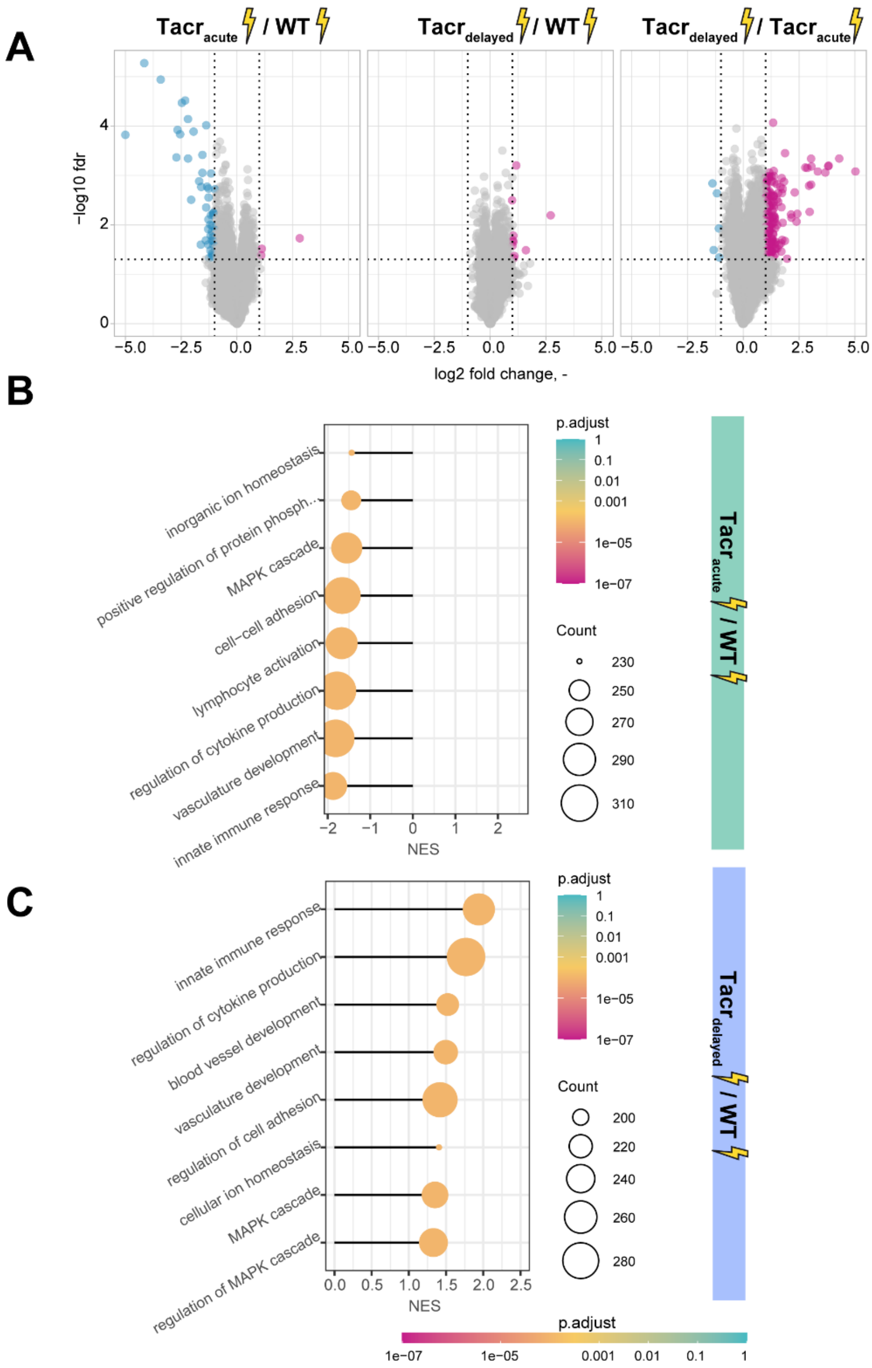

Suppl. Fig. 6: Gene expression differences between acute and delayed Tacrolimus treatment after stroke. (A)) Volcano plot of stroked WT-Tacr$_{acute}$ mice to stroked WT (left) stroked WT-Tacr$_{delayed}$ mice to stroked WT (middle) and stroked WT-Tacr$_{delayed}$ mice to stroked WT-Tacr$_{acute}$ (right). **(B)** Lollipop plot and of top 10 pathways enriched in (A) stroked WT-Tacr$_{acute}$ mice to stroked WT and (B) stroked WT-Tacr$_{delayed}$ mice to stroked WT. Size of dots represents the number of genes in the pathway and color of dots represents adjusted p value.
